# Pharmacological and nutritional targeting of voltage-gated sodium channels in the treatment of cancers

**DOI:** 10.1016/j.isci.2021.102270

**Published:** 2021-03-06

**Authors:** Osbaldo Lopez-Charcas, Piyasuda Pukkanasut, Sadanandan E. Velu, William J. Brackenbury, Tim G. Hales, Pierre Besson, Juan Carlos Gomora, Sébastien Roger

**Affiliations:** 1 Université de Tours, EA4245 Transplantation, Immunologie, Inflammation, Faculté de Médecine de Tours, 10 Boulevard Tonnellé, 37032 Tours, France; 2 Department of Chemistry, The University of Alabama at Birmingham, CHEM 280. 901, 14th Street S, Birmingham, AL 35294, USA; 3 Department of Biology, York Biomedical Research Institute, University of York, Heslington, York YO10 5DD, UK; 4 Institute of Academic Anaesthesia, Division of Systems Medicine, School of Medicine, the University of Dundee, DD1 9SY, Dundee, UK; 5 Instituto de Fisiología Celular, Circuito Exterior s/n Ciudad Universitaria, Universidad Nacional Autónoma de México, Mexico City, 04510 México; 6 Institut Universitaire de France, 75005 Paris, France

**Keywords:** Cell Biology, Cancer

## Abstract

Voltage-gated sodium (Na_V_) channels, initially characterized in excitable cells, have been shown to be aberrantly expressed in non-excitable cancer tissues and cells from epithelial origins such as in breast, lung, prostate, colon, and cervix, whereas they are not expressed in cognate non-cancer tissues. Their activity was demonstrated to promote aggressive and invasive potencies of cancer cells, both *in vitro* and *in vivo*, whereas their deregulated expression in cancer tissues has been associated with metastatic progression and cancer-related death. This review proposes Na_V_ channels as pharmacological targets for anticancer treatments providing opportunities for repurposing existing Na_V_-inhibitors or developing new pharmacological and nutritional interventions.

## Graphical abstract

**Figure:**
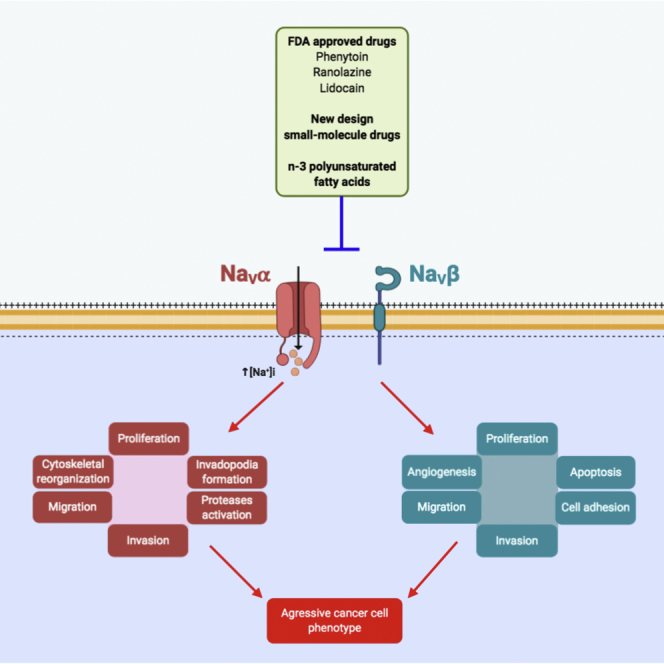

## Introduction

Voltage-gated sodium (Na_V_) channels, composed of pore-forming Na_V_α and auxiliary Na_V_β subunits, were initially characterized in excitable cells in which they are responsible for the triggering and the propagation of action potentials. Their physiological activity, through a transient depolarizing inward sodium current in cell types such as cardiomyocytes, skeletal muscle cells, or neurons, is well characterized as being responsible for the initiation of excitation-contraction, excitation-secretion, or excitation-expression couplings. As such, these ion channels are critical in numerous physiological functions and mutations in their encoding genes, as well as dysregulation of their activity may lead to serious pathologies called “sodium channelopathies.” Na_V_ channels are targets for multiple inhibitory molecules that are FDA approved and clinically used in the treatment of pathologies such as cardiac angina or arrhythmias, epilepsies, chronic pain, or in anesthesiology.

Although the activity of Na_V_ channels has been characterized about 70 years ago, recent data obtained in the past 5 years have shed light on the protein structure, arrangement, and functioning at the molecular level. Indeed, the activity of Na_V_ channels, i.e. sodium currents (I_Na_), was first recorded by Hodgkin and Huxley in 1952 from the squid giant axon, using the voltage-clamp technique. These pioneering experiments led to the ionic theory of membrane excitability (Hodgkin and Huxley, 1952). However, at that time the structure of Na_V_ channels was not known and the first evidence of molecular properties came to light at the beginning of the 1980s with the identification of the channel proteins using radiolabeled-neurotoxins highly selective for Na_V_ channels in combination with protein solubilization and purification methods (Agnew et al., 1980; Beneski and Catterall, 1980). Further structural insights into Na_V_ channels were obtained by cloning and screening of cDNA libraries leading to the discovery of the amino acid sequence for these proteins and allowing for modeling of secondary structures based on aliphatic profiles (Noda et al., 1984, 1986). These seminal studies allowed development of a model in which the pore-forming Na_V_ principal subunit in eukaryotes, later called Na_V_α-subunit, is composed of a single polypeptide chain of approximately 260 kDa containing four repeated homologous domains (I–IV) of six transmembrane segments (S1–S6). This protein was identified to interact with one or two single-span transmembrane auxiliary subunits, called Na_V_β-subunits (30–40 kDa), initially characterized as bringing regulatory functions (Isom et al., 1992, 1994) to the macromolecular complex of eukaryotic Na_V_ channels (Brackenbury and Isom, 2011; Catterall, 2000).

Interestingly, understanding of protein organization and function at the molecular level substantially progressed very recently with the use of X-ray protein crystallography and cryo-electron microscopy, first studying tetrameric prokaryotic channels sharing approximately 25–30% sequence identity with human channels. Thus information regarding the voltage-dependent gating; ion selectivity; drug binding; and open, closed, and inactivated states were acquired from the *Arcobacter butzleri* (NavAb) channel (Payandeh et al., 2011, 2012) (Lenaeus et al., 2017). The high-resolution crystal structure of the complete voltage-gated sodium channel (Na_V_Ms) from *Magnetococcus marinus* was obtained in the activated open state, associated with electrophysiological recordings (Sula et al., 2017). Recently, structural data obtained for Na_V_Ab provided a complete gating mechanism for voltage sensor function, pore opening, and the activation gate (Wisedchaisri et al., 2019). The first near-atomic resolution structure of a monomeric eukaryotic channel came from the recent study of the Na_V_PaS from the American cockroach, whereas structures of human voltage-gated sodium channel Na_V_1.2, Na_V_1.4, and Na_V_1.7, in complex with β-subunits were recently published (Pan et al., 2018, 2019; Shen et al., 2019). These structural data provide important insight into mechanisms underlying Na_V_ channelopathies and for drug discovery. Briefly, each domain presents two functional modules: S1–S4 segments comprise the voltage-sensor module (VSM), whereas sections (S5 - P loop - S6) constitute the pore-forming module (PM). The positively charged arginine and lysine residues, positioned at every third residue within each S4 segment in the voltage-sensor module, sense changes in the transmembrane potential and transform this electrical stimulus into a fast conformational change of the pore-forming module, allowing the opening of the conductive pore, permitting Na^+^ influx. One to two milliseconds after pore opening, another fast voltage-dependent mechanism happens in the Na_V_ channel protein, occluding Na^+^ influx, a process known as fast inactivation.

Another recent study has challenged the initial paradigms by which functional channels contain a monomeric Na_V_α subunit. Indeed, Na_V_α-subunits appear to assemble as dimers, and this physical interaction permits a coupled gating mechanism (Clatot et al., 2017). In humans, there are nine different genes encoding for Na_V_α-subunits, four of them clustered on chromosome 2: *SCN1A* (Na_V_1.1), *SCN2A* (Na_V_1.2), *SCN3A* (Na_V_1.3), and *SCN9A* (Na_V_1.7); three others located on chromosome 3: *SCN5A* (Na_V_1.5), *SCN10A* (Na_V_1.8), and *SCN11A* (Na_V_1.9); and two more located on chromosome 12 and 17: *SCN8A* (Na_V_1.6) and *SCN4A* (Na_V_1.4), respectively (Goldin, 2002). The amino acid sequence homology among Na_V_ channels subtypes is higher than 70% in the transmembrane and extracellular motifs so that there are no distinct subfamilies; nonetheless, some of the isoforms are more closely related to each other, sharing chromosomal localization and sensitivity to tetrodotoxin (TTX), which has been explained by the early genomic duplication during the evolution of Na_V_ channel genes. To date, four genes have been identified for Na_V_β-subunits, one on chromosome 19: *SCN1B* (encoding for the two splicing variants, transmembrane Na_V_β1 and soluble Na_V_β1B) and the other three on chromosome 11: *SCN2B* (Na_V_β2), *SCN3B* (Na_V_β3), and *SCN4B* (Na_V_β4). The structure of Na_V_β-subunit is comprised of an N-terminal extracellular immunoglobulin-like domain, followed by an extracellular juxtamembrane region, a single transmembrane segment and a 34-44 amino-acid-length intracellular domain, except for the splice variant Na_V_β1B, which is expressed as a soluble macromolecule (Brackenbury and Isom, 2011). Furthermore, it is worth noting that Na_V_β-subunits not only influence Na_V_α-subunit trafficking and biophysical modulation but they have also been experimentally shown to act as cell adhesion molecules (CAMs), participating in both homophilic and heterophilic interactions, with contactin, N-cadherin, NrCAM, and several types of neurofascin and tenascin being the main binding proteins (Isom, 2002; Bouza and Isom, 2018).

Na_V_α and Na_V_β subunits are differentially and developmentally expressed in several tissues and cell types (Black and Waxman, 2013; Roger et al., 2015). Initial studies identified Na_V_ to be distributed in excitable tissues such as in mammalian central and peripheral nervous systems as well as in skeletal and cardiac muscle. The central nervous system channels mainly comprise the Na_V_1.1, Na_V_1.2, Na_V_1.3, and Na_V_1.6 isoforms, whereas the peripheral nervous system channels include Na_V_1.7, Na_V_1.8, and Na_V_1.9. Na_V_1.4 and Na_V_1.5 were characterized as being the main skeletal and heart muscle isoforms, respectively (Goldin, 2001), whereas they also have been identified to be expressed in the brain and in the dorsal root ganglion (Wang et al., 2008, 2009, 2018a, 2018b; Bergareche et al., 2015). Therefore, the expression of Na_V_ has long been considered as the hallmark of excitable cells. Again, this paradigm has recently changed with the identification of expression (mRNA and protein) and sometimes activity at the plasma membrane (transient sodium currents) in non-excitable tissues and cells such as in chondrocytes, endothelial cells, microglia, astrocytes, fibroblasts, keratinocytes, islet β-cells, red blood cells, T-lymphocytes, dendritic cells, and macrophages, among others, in which the biological role and subcellular distribution of Na_v_s is still elusive (Black and Waxman, 2013; Roger et al., 2015).

Recently, it has emerged that Na_V_α channels, as well as auxiliary Na_V_β subunits, are aberrantly expressed in non-excitable cancer tissues and cells from different epithelial origins such as breast, lung, prostate, colon, and cervix, whereas they are not expressed in cognate non-cancer tissues. Their expression in carcinoma cells has been associated with cancer progression, suggesting that they could serve as cancer markers and prognostic factors. The expression and activity of Na_V_ channels was shown to promote pro-cancerous properties and, importantly, to contribute to disease progression, in both *in vitro* and *in vivo* models. Recent studies shed light on the signaling pathways that are under the control of these channel proteins, coupling membrane activity to cellular properties. Furthermore, the inhibition of Na_V_ channel activity, using small synthetic compounds and FDA-approved drugs as well as with natural dietary compounds potentially opens up new therapeutic strategies. In this review, we summarize current knowledge on the expression of Na_V_ channels in cancers, highlight the signaling pathways involved, and discuss pharmacological and nutritional strategies that represent opportunities for novel anticancer treatments.

### The aberrant expression of Na_V_α and Na_V_β subunits in cancers

**Figure 1:**
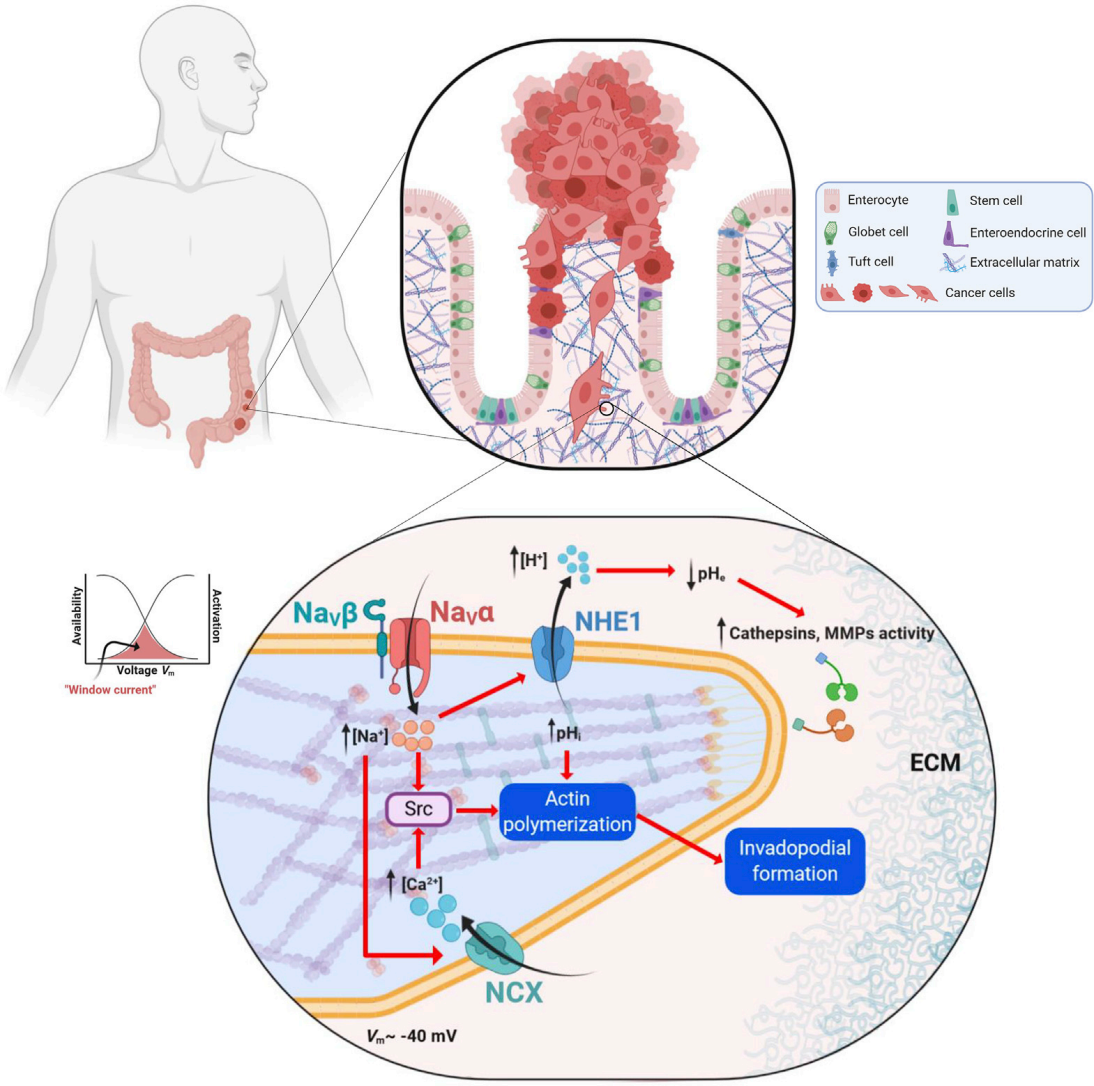
Expression of Na_V_α in carcinoma and role in invadopodial activity and invasion of extracellular matrices Progression of precancerous into cancer cells is illustrated in the context in the malignant transformation of colon epithelium. Transformed cells have lost cell polarity, replication control, and cell-cell adherent junctions, and they acquired a mesenchymal pro-invasive phenotype. Migrating cancer cells develop a specialized actin-based membrane protrusions called “invadopodia” that facilitate cell invasion by providing a coupling of focal extracellular matrix (ECM) degradation together with a directional cell movement. Na_V_ channels are expressed in invadopodial structures, co-localizing with the Na^+^/H^+^ exchanger type 1 (NHE1). Activity of Na_V_ channels enhances the extrusion of protons by NHE1 and therefore the acidification of the peri-invadopodial microenvironment, thus favoring both secretion and activity of ECM proteases such as cysteine cathepsins and matrix metalloproteinases (MMPs). Cancer cell resting potential (*V*_*m*_) is around −40 mV, in a window of voltage of Na_V_ channels (overlap between activation and steady-state inactivation curves) in which a small proportion of channels are activated but non-inactivated, thus generating a small but continuous Na^+^ influx through a so-called “window sodium current.” Na_V_ channels are also proposed to increase the intracellular levels of Ca^2+^ ions by the functioning of Na^+^/Ca^2+^ exchanger (NCX) in a “reverse mode.” Thus, the increase in the intracellular concentration of Na^+^ and Ca^2+^, sustains SRC kinase activity, leading to the polymerization of acting filaments and the formation of invadopodial structure.

The expression of voltage-gated sodium channel subunits, both Na_**V**_α and Na_**V**_ β, has been reported to be altered in several types of cancer (Table 1). The channel subunits have been detected by molecular biology and biochemical techniques, and in multiple cancer types Na_V_ activity at the plasma membrane, i.e. I_Na_, could be recorded. Most subunits have been shown to be upregulated in cancers, whereas some of them appear to be downregulated. This aberrant expression has been correlated with oncogenic properties in both *in vitro* and *in vivo* models of cancer, mostly in solid tumors including carcinomas (Figure 1).

**Table 1:** Expression and identified roles of pore-forming Na_V_α and auxiliary Na_V_β in cancer

Isoform	Expression levels	Cancer type	Role in cancer, proposed mechanism	References
Na_V_1.1	Upregulated^a^ (mRNA, protein)	Lymph nodes from CRC	Unknown, unknown	Lin et al. (2019)
Na_V_1.2	Upregulated^a^ (Protein)	Liver	Unknown, unknown	The Human protein Atlas (www.proteinatlas.org)
Na_V_1.3	Upregulated^a^ (mRNA)	Ovarian	Unknown, unknown	Gao et al. (2010)
Na_V_1.4	Upregulated^a^ (mRNA)	Cervix	Unknown, unknown	Diaz et al. (2007)
Na_V_1.5	Upregulated^a^^,^^b^ (mRNA, protein, I_Na_^+^)	Breast	↑Invasion^c^^,^^d^ ▪ Through an increased Src kinase activity promoting invadopodial formation and favoring an allosteric activation of NHE-1, extracellular acidification and enhanced activity of cysteine−cathepsins proteases ▪ Boosting the EMT transition via SIK1 ▪ Generating a sustained plasma membrane depolarization that leads to Rac1 activation and cytoskeleton reorganization ▪ Increasing MMP9 expression and reducing apoptosis	Roger et al. (2003), Fraser et al. (2005), Brackenbury et al. (2007), Gillet et al. (2009), Brisson et al. (2011), Yang et al. (2012), Brisson et al. (2013), Driffort et al. (2014), Nelson et al. (2015a),b, Gradek et al. (2019), Yang et al. (2020),
		Colorectal	↑Invasion^c^ Through the regulation of the transcriptional pathway PKA/ERK/c-jun/ELK-1/ETS-1	House et al. (2010), Baptista-Hon et al. (2014), House et al. (2015), Guzel et al. (2019), Poisson et al. (2020)
		Ovarian	↑Migration^c^ ↑Invasion^c^ ↑Proliferation^c^ ▪ By increasing the window current and depolarization of resting potential	Gao et al. (2010), Liu et al. (2018)
		Leukemia	↑Invasion^c^ ▪ Endosomal acidification and enhanced phagocytosis via calcium signaling	Fraser et al. (2004), Carrithers et al. (2007), Carrithers et al., 2012, Rahgozar et al. (2013), Jones et al. (2014), Huang et al. (2015)
Na_V_1.6	Upregulated^a^^,^^b^ (mRNA, protein, I_Na_^+^)	Cervix	↑Invasion^c^ ▪ Boosted activity of MMP2 and NHE-1	Diaz et al. (2007), Hernandez-Plata et al. (2012), Lopez-Charcas et al. (2018)
		Leukemia	↑Invasion^c^ ▪ Invadopodial formation and enhanced activity	Carrithers et al. (2009)
		Melanoma	↑Invasion^c^ ▪ Invadopodial formation and enhanced activity	Carrithers et al. (2009)
		Lymph nodes from CRC	Unknown, unknown	Lin et al. (2019)
	Downregulated^a^^,^^b^ (mRNA, protein, I_Na_^+^)	Erwin sarcoma	↓Migration^c^ ↑Apoptosis^c^ Through a repression of *SCN8A* by RING1B and maintaining low NF-κβ levels	Hernandez-Munoz et al. (2016)
		Colorectal	Unknown, unknown	Igci et al. (2015)
Na_V_1.7	Upregulated^a^^,^^b^ (mRNA, protein, I_Na_^+^)	Prostate	↑Invasion^c^^,^^d^ ▪ Cell motility increased via galvanotaxis	Grimes et al. (1995), Diss et al. (2001), Diss et al. (2005), Yildirim et al. (2012), Bugan et al. (2019)
		Lung (NSCLC)	↑Invasion^c^ ▪ Dysregulation of sodium homeostasis ▪ Increase of [NA^+^]_i_ and depolarization of cell membrane	Roger et al. (2007) Campbell et al. (2013)
		Lung (SCLC)	Unknown Participation in the formation of a neuroendocrine-like tumor cell phenotype	Pancrazio et al. (1989), Blandino et al. (1995), Onganer et al. (2005)
		Stomach	↑Invasion^c^^,^^d^ ↑Tumor growth^d^ ▪ Cancer progression through MACC1-mediated upregulation of NHE-1	Xia et al. (2016)
		Endometrial	↑Invasion^c^^,^^d^↑Tumor growth^d^↓Apoptosis^d^ ▪Unknown	Liu et al. (2018), Liu et al. (2019)
	Downregulated^a^ (mRNA, protein)	Colorectal	Unknown, unknown ▪ Proposed within a risk score with high prognostic value for overall survival	Pan et al. (2017)
Na_V_1.8	Upregulated^b^ (mRNA)	Lung (NSCLC)	Unknown, unknown	Roger et al. (2007)
Na_V_1.9	Upregulated^b^ (mRNA)	Lung (NSCLC)	Unknown, unknown	Roger et al. (2007)
Na_V_β1	Upregulated^a^^,^^b^ (mRNA, protein)	Prostate	Unknown, unknown	Diss et al. (2008)
		Breast	↑Tumor growth^c^^,^^d^ ↑Vascularization^d^ ↑Invasion^c^ ↓Apoptosis ▪ β1 *trans-homophilic adhesion* triggering of cell process outgrowth via Fyn kinase	Nelson et al. (2014), Bon et al. (2016)
	Downregulated^b^ (mRNA, protein)	Breast	↑Invasion^c^ By decreasing cell adhesion and facilitating cell migration	Chioni et al. (2009)
		Lung	↑Invasion^c^ By decreasing cell adhesion	Campbell et al. (2013)
		Cervix	↑Migration^c^ ▪Acting as a cell adhesion molecule ↓Proliferation^c^ ▪By increasing the population of cells in the G0/G1 phase in cell cycle	Sanchez-Sandoval and Gomora (2019)
Na_V_β2	Upregulated^b^ (mRNA, protein)	Prostate	↑Invasion^c^ ▪ Promotion of bipolar cell morphology, enhanced cell adhesion and motility through association with neural substrates. ↓Tumor volume in xenograft models^‡^	Jansson et al. (2012), Jansson et al. (2014)
Na_V_β3	Upregulated^b^ (mRNA, protein)	Bone	↑Apoptosis^c^ ▪ Increase of a p53-dependent apoptotic pathway	Adachi et al. (2004)
Na_V_β4	Downregulated^a^^,^^b^ (mRNA, protein)	Breast	↑Invasion^c^^,^^d^ ▪ Enhanced RhoA activity ▪ Mediated by the intracellular C-terminus motif ▪ Acquisition of a hybrid mesenchymal-amoeboid aggressive phenotype	Bon et al. (2016)
		Lung	Unknown, unknown	Bon et al. (2016)
		Cervix	↑Invasion^c^	Sanchez-Sandoval and Gomora (2019)
		Thyroid	Unknown ▪ Its expression is an indicator of favorable recurrence-free survival	Gong et al. (2018)

a Cancer tissue versus non-cancerous tissue.

b Highly invasive cell line versus normal immortalized cell line.

c *In vitro*.

d *In vivo*.

#### Na_V_α and Na_V_β subunits in prostate cancer

Although early works assessing ion channel activity identified sodium currents in small-cell lung cancer cells (Pancrazio et al., 1989), the first report of the direct contribution of Na_**V**_ channels in cancer properties came from work performed in prostate cancer (PCa) cells by Prof. M. Djamgoz’ group almost 25 years ago. This first study was performed in two rat prostatic tumor cell lines in which they found a differential expression of voltage-activated Na^+^ currents: highly metastatic Mat Ly-Lu cells expressed Na^+^ currents, whereas weakly metastatic AT-2 cell did not express this type of current. For the first time, the functional relevance of the Na^+^ current was demonstrated: blocking I_Na_ with nanomolar concentrations of TTX reduced by ∼30% the invasiveness of the highly metastatic Mat-Ly-Lu cells (Grimes et al., 1995). Further evidence showed that the Na_**V**_1.7 pore-forming subunit, encoded by the *SCN9A* gene, was overexpressed in highly metastatic human and rat prostate cell lines in comparison with weakly metastatic cell lines (Diss et al., 2001). Later, this observation was confirmed *in vivo* by showing that Na_**V**_1.7 was overexpressed approximately 20 times in PCa biopsies versus non-cancer samples (Diss et al., 2005). Finally, in a rat model, inoculation with highly metastatic Mat-Ly-Lu cells promotes prostate cancer metastasis *in vivo*, and blockade of Na_**V**_ in primary tumors with TTX (Yildirim et al., 2012) or ranolazine (Bugan et al., 2019) reduced lung metastases by 40% and 63%, respectively. However, most of these studies were performed in cell lines, and there are no systematic studies that show a positive correlation between mRNA/protein of Na_**V**_1.7 upregulation and human PCa patient samples.

Concerning Na_**V**_β subunits, it was shown that Na_**V**_β1 mRNA was the most highly expressed in strongly invasive compared with weakly invasive PCa cell lines (Diss et al., 2008). However, there was no significant difference in the expression of Na_**V**_β1, or any other Na_**V**_β subunits, between cancer and non-cancer human prostate specimens (Diss et al., 2008). Na_**V**_β2 has been proposed to participate in prostate cancer biology. Indeed, the first experimental observation showed that the overexpression of Na_**V**_β2, fused to GFP, was properly localized at the plasma membrane of weakly metastatic LNCaP cells inducing morphological changes consistent with a bipolar and elongated migratory phenotype (Jansson et al., 2012). These changes were accompanied by an increase in cell migration and Matrigel invasion *in vitro*, although there was no significant change in cell proliferation. However, the *in vivo* properties of LNCaP cells overexpressing the Na_**V**_β2 subunit were quite unexpected, as subcutaneous tumor volume was drastically reduced compared with the control group (Jansson et al., 2012). Such contrasting effects could imply different mechanisms involved in tumor formation, growth, and metastatic behavior. A novel *ex vivo* organotypic spinal cord co-culture with LNCaP cells revealed that overexpression of Na_**V**_β2 enhanced the association of PCa cells with nerve axons (Jansson et al., 2014). Furthermore, overexpression of Na_**V**_β2 enhanced PCa cell migration, invasion, and growth, in the presence of the neuronal CAM laminin, suggesting that the Na_**V**_β2 subunit may mediate metastatic behavior through association with neural substrates (Jansson et al., 2014).

#### Na_V_α and Na_V_β subunits in breast and colorectal cancer

Breast cancer (BCa) is the most lethal female cancer worldwide (Bray et al., 2018), and colorectal cancer (CRCa) is the third most commonly diagnosed cancer (Arnold et al., 2017). The incidence of these cancers is gradually increasing, thus representing a serious global health problem. The main cause of patient mortality from these two types of cancers, as for the majority of carcinomas, is the development of metastases in distant organs, following the dissemination of cancer cells from the primary tumor (Figure 1).

Multiple studies have investigated the expression of Na_V_ channels and their contribution to tumor progression and metastasis in BCa and CRCa. Knowledge about signaling pathways and cellular mechanisms induced by Na_V_ channels have been mostly acquired from these cancers. In both cases, the major isoform identified was Na_**V**_1.5, encoded by the *SCN5A* gene. In BCa samples, Na_V_1.5 is overexpressed as compared with normal tissues (Fraser et al., 2005). A high expression was correlated with cancer recurrence, metastasis development, and reduced patient survival (Yang et al., 2012). Most of the mechanistic studies in BCa have been performed in human cancer cell lines such as MDA-MB-231 (highly metastatic) and compared with weakly metastatic cell lines such as MCF-7. It has been shown initially that MDA-MB-231 expresses a TTX-resistant Na^+^ current, lacking in MCF-7 cells (Roger et al., 2003), which is encoded by a neonatal splice variant of the *SCN5A* gene (Fraser et al., 2005). This neonatal variant is due to a switch from adult exon 6B to fetal exon 6A, which are mutually exclusive and encode for a part of the voltage sensor, segments 3 and 4 located in the domain I of the channel. Therefore, these two variants, called hNa_V_1.5 and hNa_V_1.5e for the adult and the neonatal channels respectively, show different electrophysiological properties in terms of voltage sensitivity and current kinetics (Murphy et al., 2012; Onkal et al., 2008). These changes result in a greater Na^+^ influx for neonatal hNa_V_1.5e (Onkal et al., 2008). In the heart, splicing of *SCN5A* is developmentally regulated, such that the neonatal exon 6A is rapidly replaced by the “adult” exon 6B after birth (Murphy et al., 2012) and molecular determinants explaining the abnormal expression of hNaV1.5e in cancer cells have not been identified so far. Nevertheless, the inhibition of channel activity, by either pharmacological (TTX, ranolazine and phenytoin) or molecular (siRNA and inhibitory antibody) approaches, has shown its contribution to migration and invasion of BCa cell lines (Roger et al., 2003; Fraser et al., 2005; Brackenbury et al., 2007; Driffort et al., 2014; Yang et al., 2012). There has also been some significant progress into uncovering the mechanisms underlying the promotion of invasiveness behavior by Na_**V**_1.5. Experimental evidence suggested that Na_**V**_1.5 induces pro-migratory and pro-invasive properties through a persistent activity at the membrane potential called “window current,” and a correlated depolarization of the membrane voltage of breast cancer cells. Particularly, Na_V_1.5 activity induced the allosteric modulation of the Na^+^-H^+^ exchanger type 1 (NHE-1), resulting in an increased activity, leading to the acidification of the extracellular space, thus favoring the pH-dependent activity of proteolytic cysteine cathepsins (Gillet et al., 2009; Brisson et al., 2011). In addition, Na_**V**_1.5 expression and activity were demonstrated to increase Src kinase activity, which promotes the acquisition of an invasive morphology (invadopodia) in MDA-MB-231 cells. Taken together, these observations indicate that Na_**V**_1.5 promotes invadopodia activity of breast cancer cells and the invasion of the surrounding ECM (Brisson et al., 2013) (Figure 1). Recently, Na_V_1.5 was identified as importantly promoting the epithelial-to-mesenchymal transition (EMT) and cancer cell invasiveness through the regulation of the salt-inducible kinase 1 (SIK1) (Gradek et al., 2019). Furthermore, Na_V_1.5 activates the small GTPase Rac1 by sustaining a plasma membrane depolarization, which as a regulator of activation, induces cytoskeletal reorganization and cellular migration (Yang et al., 2020). In addition, *in vivo* experiments have shown that Na_**V**_1.5 activity promotes metastasis in immunodeficient mice (Driffort et al., 2014; Nelson et al., 2015a, 2015b). Na_V_1.5 activity also increases MMP9 expression and reduces apoptosis in primary tumors *in vivo* (Nelson et al., 2015b).

**Figure 2:**
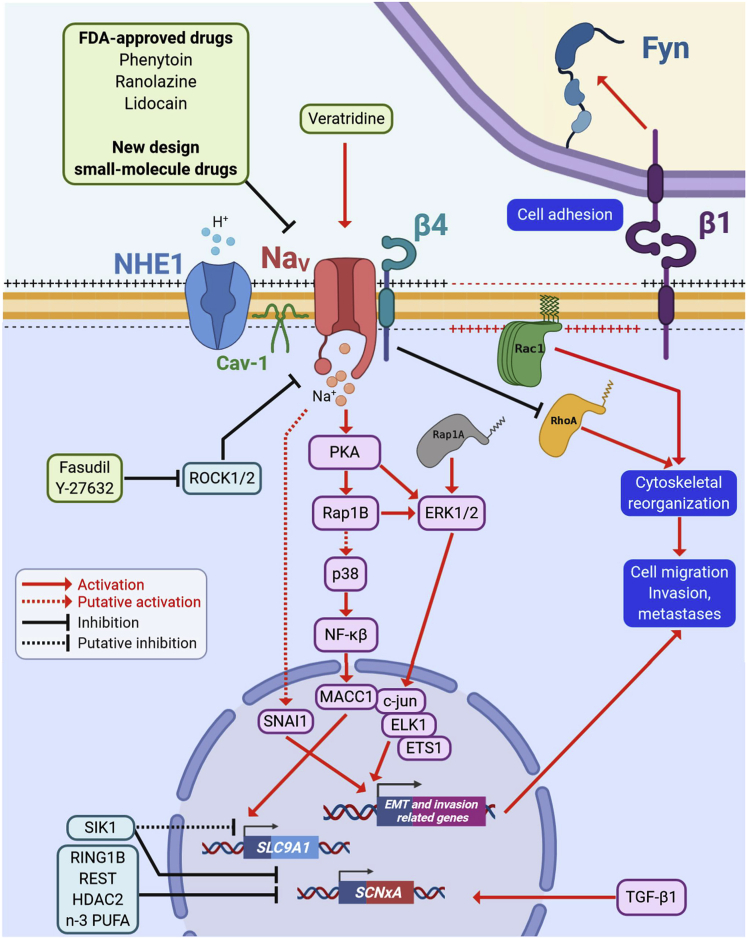
Participation of Na_V_α and/or Na_V_β in pro-metastatic signaling pathways Na_V_α subunit overexpression and activity in cancer cells trigger biochemical or an electro-biochemical cascades, leading to the acquisition of a pro-invasive cell phenotype. Na_V_ is co-localized with NHE1 in caveolin-1 (Cav-1)-containing lipid rafts and promotes the efflux of protons. Na_V_ activity can be further stimulated by the use of pharmacological activators such as veratridine (inhibitor of the inactivation phase). Activity of Na_V_α subunits leads to a cAMP-independent activation of protein kinase A (PKA) that activates the cytosolic small GTPase Ras-related protein 1 (Rap1A/B) and the extracellular-signal-regulated kinases (ERK1/2). The transcription factor (TF) metastasis associated in colon cancer 1 (MACC1) is activated by the p38/NF-κβ signaling, whereas the TFs c-jun, ELK1, and ETS1 are activated by ERK1/2 and the zinc finger protein SNAI1 is activated through a Na_V_α-dependent mechanism regulating the expression of genes associated with cytoskeleton reorganization, cell motility, extracellular matrix degradation, and cell invasiveness. It has been demonstrated that MACC1 upregulates the expression of the *SLC9A1* gene, encoding for NHE1, thus enhancing its activity at plasma membrane. On the other hand, the electro-biochemical triggering begins with a resting potential depolarization due to the activity of Na_V_α subunits promoting the activation and recruitment of the small GTPase Ras-related C3 botulinum toxin substrate 1 (Rac1) at the leading edge of migrating cells. Transforming growth factor β 1 (TGF-β1) increases the expression levels of Na_V_ channels genes (*SCNxA*), whereas ring finger protein 1 (RING1B), RE1 silencing transcription factor (REST), histone deacetylase 2 (HDAC2) and salt inducible kinase 1 (SIK-1), as well as the n-3 polyunsaturated fatty acids n-3 (PUFA) repress their expression. SIK-1 also impairs the functioning of NHE1 exchanger. The “auxiliary subunit” Na_V_β4 is expressed in normal epithelial cells but is importantly downregulated in invasive cells and high-grade metastatic tumors. The absence of this protein, but specifically the lack of the intracellular C-terminus domain, triggers the acquisition of an amoeboid-mesenchymal hybrid phenotype dependent of the small GTPase Ras homolog family member A (RhoA). Na_V_β1 proteins have a dual role in cancer cells acting as cell adhesion molecules (CAMs), reducing cell migration and proliferation. However, it has also been demonstrated that Na_V_β1 promotes tumor growth, metastasis, and vascularization via the proto-oncogene tyrosine-protein kinase Fyn. The Rho-associated protein kinases (ROCK1/2) negatively regulate the expression of Na_V_α subunits, therefore, silencing or inhibition of these repressors restore Na_V_ channels activity promoting an aggressive cell phenotype. Pharmacological intervention with FDA-approved drugs or new-design small-molecule lead compounds against Na_V_ channels represents a promising strategy to decrease sodium-channel-associated metastases.

The *SCN5A* gene and its protein product the Na_**V**_1.5 channel have also been shown to be overexpressed in colorectal cancer biopsies, as compared with non-cancer samples (House et al., 2010). Na_V_1.5 was found to be expressed at the plasma membrane of tumor cells, and its activity (I_Na_) was recorded in several carcinoma cell lines (mainly SW-480, SW-620, and HT-29) (House et al., 2010). In colon cancer cells, Na_V_1.5 activity promotes cancer cell invasion *in vitro*, in both 2- and 3-dimensional models, and regulates a network of invasion-promoting genes via modulation of the PKA/ERK/c-JUN/ELK-1/ETS-1 transcriptional pathway (House et al., 2010, 2015; Poisson et al., 2020) (Figure 2). It was shown that, similar to BCa, the neonatal exon 6A splice variant of the Na_**V**_1.5 isoform has a predominant contribution to the invasiveness of CRCa cell lines (Guzel et al., 2019), even though both adult hNa_V_1.5 and neonatal hNa_V_1.5e splice variants could be detected (Baptista-Hon et al., 2014).

A study reported the downregulation of the *SCN9A* gene, encoding for Na_V_1.7, in CRCa (Pan et al., 2017). In this study, authors analyzed genes differentially expressed in CRCa utilizing three Gene Expression Omnibus (GEO) datasets. By screening 46 biomarkers associated with cancer proliferation, drug-resistance, and metastasis, i.e., genes closely associated to patient overall survival, they proposed a risk score with high prognostic value based on the expression of five genes: *MET* (MET proto-oncogene and receptor tyrosine kinase), *CPM* (carboxypeptidase M), *SHMT2* (serine hydroxymethyltransferase 2), *GUCA2B* (guanylate cyclase activator 2B), and *SCN9A*. *MET* and *SHMT2* were upregulated whereas *CPM*, *GUCA2B*, and *SCN9A* were downregulated. Interestingly, this observation was confirmed in the human protein atlas immunohistochemistry database (www.proteinatlas.org), as the staining for Na_**V**_1.7 was lower in some CRCa sample tissues (Pan et al., 2017). There are also reports indicating the downregulation of Na_**V**_1.6, encoded by the *SCN8A* gene, in CRCa (Igci et al., 2015). Tumor samples from CRCa patients exhibited reduced expression of Na_**V**_1.6 compared with paired tumour-surrounding normal tissues. *SCN8A* mRNA levels, analyzed by real-time qPCR, were significantly lower in tumor tissues and in patients younger than 45 years. Results also reveal a relationship between *SCN8A* expression, gender, grade of CRCa, tumor location, and histopathological classification (Igci et al., 2015). On the contrary, Na_V_1.6 protein was highly expressed in metastatic lymph nodes from CRCa patients (Lin et al., 2019). Although the reduced expression of *SCN8A*, encoding for Na_V_1.6, and *SCN9A*, encoding for Na_V_1.7, might harbor predictive values in CRCa, we are still missing clear information to assert whether they have a role, either causative or consecutive, in the carcinogenesis or whether their expression dysregulation is only correlative to cancer transformation or progression. Indeed, the functional activity of Na_V_1.6 and/or Na_V_1.7 at the plasma membrane of colorectal non-cancer or cancer cells has not been demonstrated so far. Furthermore, it cannot be excluded that these channels might be expressed in intracellular compartments, in which they might play diverse functions. Eventually, it is not clear at the moment whether these changes in expression levels concern epithelial cells, or non-epithelial cells in the colorectal tract, such as immune cells, which are key protagonists in colorectal carcinogenesis. As such, the participation of *SCN8A* and *SCN9A* in CRCa biology will require further studies.

The expression of Na_**V**_β subunits has been studied in BCa and in CRCa. Some Na_**V**_β have been shown to be upregulated, whereas others are downregulated in cancer tissues, and mostly these changes appear to correlate with the metastatic behavior of cancer cells, in particular with cell migration and invasion. Most research performed so far studying the role of Na_**V**_β subunits in metastatic behavior has been undertaken in BCa. Originally, it was shown that Na_**V**_β1 was more abundantly expressed in the weakly metastatic MCF-7 than in the highly metastatic MDA-MB-231 cell line. Interestingly, when MCF-7 cells were transfected with specific siRNA directed against Na_**V**_β1, cell adhesion was reduced by 35%, whereas migration was increased by 121%. In contrast, stable expression of Na_**V**_β1 in MDA-MB-231 cells increased process length and adhesion while reducing lateral motility and proliferation. Thus, Na_**V**_β1 was proposed to act as a cell adhesion molecule in BCa cells, negatively controlling cellular migration (Chioni et al., 2009). Later, it was found that Na_**V**_β1 was the Na_V_β subunit most expressed in BCa and was upregulated (both mRNA and protein) in BCa biopsies, compared with normal breast tissue (Nelson et al., 2014; Bon et al., 2016). More importantly, by using a xenograft model of BCa, it was shown that Na_**V**_β1 overexpression increased tumor growth, metastasis, and vascularization, while decreasing apoptosis in the primary tumors. Therefore, this study was the first showing the functional role for Na_**V**_β1 in tumor growth and metastasis *in vivo* (Nelson et al., 2014). Consistent with these results, the use of siRNA to specifically target Na_**V**_β1 expression in MDA-MB-231 cells inhibited cancer cell invasion (Bon et al., 2016).

The participation of Na_**V**_β3 in tumorigenesis process is poorly understood. Two missense mutations have been identified in the *SCN3B* gene in high-grade metastatic colorectal cancer biopsies (Sjoblom et al., 2006). The first report suggested that non-mutated Na_V_β3 mediates a p53-dependent apoptotic pathway in Saos-2, a bone osteosarcoma cell line, after DNA damage (Adachi et al., 2004). In agreement with this, the *SCN3B* gene is not expressed in highly invasive MDA-MB-231 breast cancer cells (Gillet et al., 2009) or weakly invasive MCF-7 cells (Chioni et al., 2009). In non-tumour breast samples, *SCN3B* expression was the lowest among all Na_V_β encoding genes and was still significantly reduced in cancer samples (Bon et al., 2016).

The *SCN4B* gene was shown to play a critical role as a metastasis-suppressor gene in BCa (Bon et al., 2016). In this study *SCN4B* mRNA appeared to be significantly expressed in normal breast, colon, rectum, lung, and prostate but consistently downregulated in cancer samples. Furthermore, Na_**V**_β4 protein was expressed in normal epithelial cells but significantly reduced in BCa biopsies, especially in high-grade primary and metastatic tumors. *In vitro* experiments showed that reducing Na_**V**_β4 expression potentiates cell migration and invasiveness though an increase in RhoA activity and the acquisition of a hybrid mesenchymal-amoeboid aggressive phenotype. This effect was independent of Na_**V**_α channel activity and was prevented by overexpression of the intracellular C-terminus of Na_**V**_β4. On the contrary, *SCN4B* overexpression reduced cancer cell invasiveness and tumor progression. The findings are in line with previous observations showing decreased levels of *SCN4B* in invasive versus non-invasive PCa cells (Diss et al., 2008). Interestingly, a recent study identified dysregulated miRNA in CRCa and reported an increased miR-424-5p expression in tumor samples that was associated with poor prognosis (Dai et al., 2020). miR-424-5p was found to be elevated in the peripheral blood of CRCa patients, most probably secreted in tumor exosomes. In this study, it was demonstrated that overexpression of *SCN4B* inhibited HT-29 CRCa cell proliferation, migration, and invasion, and expression of *SCN4B* was directly inhibited by miR-424-5p (Dai et al., 2020). These results support the tumour-suppressor role of *SCN4B* in CRCa and identified miR-424-5p as a regulator of its expression in tumors.

#### Na_V_α and Na_V_β subunits in lung cancer

There are two subtypes of lung cancer, small-cell lung cancer (SCLC) and non-small cell lung cancer (NSCLC). Early works assessing ion channel activity have been undertaken in small-cell lung cancer cells. In these pioneering works, I_Na_ was initially recorded in human H146, H69, and H128 small-cell lung cancer cell lines (Pancrazio et al., 1989), although this study did not relate the presence of Na_**V**_ currents to lung tumorigenesis process itself. These currents were most probably due to TTX-sensitive channels, because they were fully inhibited by 5 μM TTX. Although neither the molecular characterization of the channels nor their biological role was described, they were proposed to participate in a “neuroendocrine-like” tumor cell phenotype (Pancrazio et al., 1989). Later, it was shown that these sodium currents were actually able to participate in the generation of action potentials in H146 SCLC cells, thus supporting this idea. Interestingly, 5 μM TTX abolished these action potentials, which implies the contribution of a TTX-resistant current (Blandino et al., 1995). In fact, H146 cell sodium currents demonstrated an IC_50_ to TTX of 215 nM, leading the authors to indicate that Na_V_ channels were weakly TTX-sensitive. However, this concentration is too high to consider the channels to belong to the TTX-S category but too low to belong to TTX-R isoforms. The most likely explanation for this would be the expression of a population of different isoforms of Na_V_ at the plasma membrane of cells, thus leading to an apparent IC_50_ that would be intermediate between TTX-S and TTX-R. Nevertheless, the possibility that Na_V_ channels expressed in these cells are variants (splice-variants or polymorphism variants) showing specific pharmacological properties cannot be excluded.

The hypothesis of the role of Na_V_α in the acquisition of a neuroendocrine phenotype was also proposed by M. Djamgoz's team (Onganer et al., 2005). Unexpectedly, in this later study, the endocytic activity of SCLC cells was inhibited by using lower nanomolar concentrations of TTX, suggesting the participation of TTX-sensitive sodium channels in these SCLC cells. In addition, they found mRNA encoding for Na_**V**_1.3, Na_**V**_1.5, and Na_**V**_1.6 in H69, H209, and H510 cell lines. The latter also showed the additional presence of Na_**V**_1.9 mRNA. Thus, it remains to be elucidated which Na_**V**_ subunit is responsible for the generation of TTX-resistant action potentials in H146 cells (Blandino et al., 1995) and whether a TTX-resistant Na_**V**_ channels contribute to migration, invasion, or some other metastatic component, other than endocytic activity (Onganer et al., 2005), in SCLC cell lines. More studies are needed investigating the expression profile and role of Na_**V**_ channels in SCLC biopsy tissue.

Later analyses were also performed in non-small cell lung cancer (NSCLC) in which the Na_**V**_1.7 isoform was shown to potentiate cancer cell invasion (Roger et al., 2007; Campbell et al., 2013). Although different NSCLC cell lines (H23, H460, and Calu-1) express mRNA for several Na^+^ channel isoforms (Roger et al., 2007), the selective inhibition of Na_**V**_1.7 activity (using TTX) or reduction of expression (by using small interfering RNA) reduced H460 cell invasion by up to 50%. On the contrary, weakly invasive A549 cells showed no evidence of functional Na_**V**_ channels (Roger et al., 2007). In addition, exogenous overexpression of the Na_**V**_1.7 subunit was sufficient to promote TTX-sensitive invasion of these cells. Interestingly, Na_**V**_1.7 protein expression was found to be higher in cancerous compared with normal-matched human lung tissue (Campbell et al., 2013). It is worth noting that at least in one NSCLC cell line (Calu-1), expression of a TTX-resistant Na_**V**_ channel significantly contributes to the invasion capacity of this strongly metastatic cell line. However, the molecular identity of the molecular mediator of I_Na_ has not been fully characterized. Non-quantitative PCR results suggested that mRNAs encoding the three known TTX-resistant Na_**V**_ channels (Na_**V**_1.5, Na_**V**_1.8, and Na_**V**_1.9) may be more abundantly expressed in Calu-1 than in H23 and H460 cells (Roger et al., 2007). Although kinetics and TTX-sensitivity of currents recorded in Calu-1 cells suggest Na_V_1.5 activity, more studies are needed to properly identify the molecular identity of the Na_V_ channels mediating the Na^+^ current in these cells. In addition, there are currently no data correlating Na_**V**_α expression in lung cancer tissue with clinical outcome.

The expression of Na_**V**_β in LCa has been assessed in several studies but so far it is difficult to conclude a general pattern. *SCN1B* mRNA was found to be expressed in H460, Calu-1, and A549 but not in the H23 NSCLC cell lines. It was also expressed in non-cancer NL-20 and BEAS-2B cells. *SCN2B* appeared to be weakly expressed in A549 cancer and NL-20 non-cancer cells, whereas not expressed at all in H23, H460, and Calu-1 cancer cell lines. *SCN3B* mRNA was found to be expressed in all these cell types with the only exception of H460. *SCN4B* mRNA was expressed in cancer H23 and non-cancer NL-20 and BEAS-2B but not in H460, Calu-1, and A549 (Roger et al., 2007). In patient samples, *SCN4B* expression levels were downregulated in lung cancer compared with normal lung tissue, and preliminary immunohistochemical analyses in lung cancer tissue microarrays showed a tendency toward decreased protein expression in high-grade primary lung tumors and metastases (Bon et al., 2016). The role of the Na_**V**_β1 protein in cell adhesion was also proposed in human non-small cell lung cancer cell lines (Campbell et al., 2013). In this study, it was shown that the highly invasive H460 cells exhibited very low expression of all Na_**V**_β subunit mRNAs, confirming previous results (Roger et al., 2007), whereas A549 cells expressed 8-fold higher levels of Na_**V**_β1 mRNA. Accordingly, cell adhesion was 2-fold higher in A549 cells compared with H460 cells (Campbell et al., 2013). Moreover, manipulation of Na_**V**_β1 mRNA expression by using siRNA or cDNA targeting *SCN1B* in these two cell lines confirmed the contribution of this subunit in the promotion of cell adhesion and reduced invasion (Campbell et al., 2013).

#### Na_V_α and Na_V_β subunits in gastric cancer

In gastric cancer (GCa) tissue samples and in two human GCa cell lines (BGC-823 and MKN-28 cells), it was shown that the *SCN9A* gene, encoding Na_**V**_1.7, is the most abundantly expressed Na_**V**_α isoform (Xia et al., 2016). A systematic evaluation of 319 GCa tumor tissue samples by immunohistochemistry revealed a correlation of Na_**V**_1.7 expression with poor prognosis, as well as correlation with the expression of the NHE1 exchanger type 1 and the oncoprotein metastasis associated in colon cancer-1 (MACC1). In addition, Na_**V**_1.7 suppression resulted in reduced invasion and proliferation rates of GC cells and growth of GC xenografts in nude mice (Xia et al., 2016). In brief, results of this study indicate that Na_**V**_1.7 promotes GCa progression through MACC1-mediated upregulation of NHE1.

#### Na_V_α and Na_V_β subunits in cervical cancer

The product of the *SCN8A* gene, the Na_**V**_1.6 channel, has been shown to be upregulated in cervical cancer (CeCa). In a study performed by using primary cultures derived from three different patient CeCa biopsies, the presence of functional Na_**V**_ channels has been identified and I_Na_ recorded. Primary cells from CeCa biopsies expressed mRNA for different TTX-sensitive Na_**V**_α subunits: Na_**V**_1.1–1.4, Na_**V**_1.6, and Na_**V**_1.7 (Diaz et al., 2007). Among these, only the *SCN8A* gene encoding for Na_**V**_1.6 was shown to be overexpressed by about 40-fold at the mRNA level in CeCa primary cultures and biopsies in comparison with non-cancerous cervical tissue. The functional relevance of this Na_**V**_ channel was demonstrated by blocking its activity with TTX as well as with the Cn2 specific toxin, which in both cases led to a significant decrease in the invasion capacity of CeCa primary culture cells, without affecting proliferative or migratory cell behavior (Hernandez-Plata et al., 2012). This suggested a role for Na_V_1.6 in extracellular matrix degradation, and indeed Na_**V**_1.6-mediated invasiveness of CeCa cells specifically involved MMP-2 activity along with increased expression of the NHE1 exchanger (Lopez-Charcas et al., 2018). In addition, CeCa cell lines more abundantly express the mRNA for the Na_**V**_1.6 variant, which has exon 18 deleted (Δ18 variant) rather than the neonatal and adult splice variants. This variant appeared to be distributed in intracellular compartments (Lopez-Charcas et al., 2018). However, the functional relevance of the Δ18 variant to the metastatic behavior of CeCa cells remains to be elucidated. Another interesting question regarding the expression of this Δ18 variant of Na_**V**_1.6 in CeCa cells is whether it has the same function in intracellular compartments as observed in macrophages and melanoma cells in which the channel has a role in podosome formation and activity (Carrithers et al., 2009).

The role of Na_**V**_β1 as a migration suppressor gene was demonstrated in three different CeCa cell lines (HeLa, SiHa, and CaSki) in which *SCN1B* mRNA levels were around 3- to 6-fold higher than those of Na_**V**_β2, Na_**V**_β3, or Na_**V**_β4. However, differences in protein levels among the four Na_**V**_β subunits were more discrete; Na_**V**_β1 was again the most highly expressed in HeLa and CaSki cells, whereas in SiHa cells, protein levels for all Na_**V**_β were more uniform (Sanchez-Sandoval and Gomora, 2019). Previously, the same group had demonstrated that Na_**V**_β1 mRNA levels were also slightly higher in CeCa biopsies than in non-CeCa tissue (Hernandez-Plata et al., 2012). In addition, it was demonstrated that Na_**V**_β1 regulated SiHa cell proliferation, specifically by affecting the proportion of cells in the G0/G1 phase of cell cycle (Sanchez-Sandoval and Gomora, 2019). Because Na_**V**_β3 was proposed to have anti-cancer properties (Adachi et al., 2004), the effect of its expression in CeCa cells was tested. However, neither its overexpression nor its downregulation affected proliferation in CeCa cell lines, suggesting that the likely pro-apoptotic activity of Na_**V**_β3 might not be a generalized mechanism in all cancer types or cells. In this regard, it has been suggested that the p53 protein status in CeCa cell lines is under the control of the E6 protein, the main oncogene expressed as a result of human papillomavirus (HPV) infection (the most frequent risk factor for CeCa incidence) of cervical epithelial cells. The early expression of E6 protein leads to the specific ubiquitination and degradation of p53 (Scheffner et al., 1993), thereby inactivating any pro-apoptotic effect due to the Na_**V**_β3 expression in basal conditions. In line with this interpretation, *SCN3B* expression was increased almost 2-fold in CeCa biopsies when compared with non-cancer samples (Hernandez-Plata et al., 2012). Further studies are needed to fully understand the potential role of *SCN3B* as well as the mechanism involved in the pro-apoptotic effect in cancer cells. More recent observations in CeCa cell lines confirm the contribution of Na_**V**_β4 to cell invasive potential, as the downregulation of *SCN4B* leads to an increase in the percentage of invading cells in three CeCa cell lines (Sanchez-Sandoval and Gomora, 2019). However, a previous study indicated that mRNA levels for Na_**V**_β4 were not significantly different between CeCa and non-CeCa biopsies tissues (Hernandez-Plata et al., 2012).

#### Na_V_α subunits in ovarian cancer and endometrial cancers

In ovarian cancer (OCa), the Na_**V**_1.5 isoform appears to be the main Na_**V**_α subunit expressed and contributing to the migration and invasion capabilities of cancer cells (Gao et al., 2010; Liu et al., 2018); however, the splicing status of Na_**V**_1.5 in this carcinoma is currently unknown.

In endometrial cancer tissues, a recent study identified the *SCN9A* gene, encoding for the Na_V_1.7 channel, as being the most highly expressed Na_V_α subunit. Na_V_1.7 expression level was associated with tumor size, local lymph node metastasis, and 5- and 10-year survival. Pharmacological inhibition using the PF-05089771 blocker selective for Na_V_1.7 and Na_V_1.8 induced cancer cell apoptosis and reduced cancer cell invasion (Liu et al., 2019).

#### Na_V_β in papillary thyroid cancer

Recent results obtained in papillary thyroid cancer (PTC) show that *SCN4B* is downregulated at both RNA and protein level as compared with normal thyroid tissues (Gong et al., 2018). Importantly, by using databases such as the Gene Expression Omnibus (GEO) and the Cancer Genome Atlas (TCGA)-Thyroid Cancer (THCA), the authors found that *SCN4B* expression was an independent indicator of favorable recurrence-free survival (RFS) in patients with classical PTC, further contributing to the notion of the *SCN4B* as a metastases-suppressor gene (Gong et al., 2018). So far, nothing is known about the expression of Na_V_α subunits in PTC.

#### Na_V_α and Na_V_β subunits in leukemia cells

Although most results related to Na_V_ in cancer were obtained from solid tumors, predominantly carcinomas, there are also some indications that Na_V_ expression might also be dysregulated in hematological disorders such as leukemia, in which they could bear oncogenic properties. In Jurkat leukemic T cell lymphoblasts, original evidence showed that a small fraction of ∼10% displayed I_Na_ and mRNA encoding for Na_**V**_1.5, Na_**V**_1.6, and to a lesser extent Na_**V**_1.7 and Na_**V**_1.9, were detected (Fraser et al., 2004). I_Na_ was likely carried mostly by a TTX-resistant Na_**V**_ channel because an IC_50_ of ∼1 μM was measured. Importantly, invasion was reduced by 93% when the cells were treated with 10 μM TTX (Fraser et al., 2004). However, more recent data have shown that Na_**V**_1.6, Na_**V**_1.7, and Na_**V**_1.3 (in that order) are the most abundant Na_**V**_ isoforms in three acute lymphocytic leukemia cell lines, including Jurkat, MOLT-4, and BALL-1 cells, as well as in peripheral blood mononuclear cells (PBMC). In this study, I_Na_ recorded from approximately 20% of MOLT-4 cells was completely abolished by 2 μM TTX, indicative of TTX-sensitive channels. The same concentration of TTX decreased the invasion of MOLT-4 and Jurkat cells by 90% (Huang et al., 2015).

Interestingly, semi-quantitative PCR results indicated the presence of both the neonatal (18N) and the Δ18 (exon 18 skipped) isoforms of Na_**V**_1.6 channel in the THP-1 monocytic leukemia cell line (Carrithers et al., 2009). Neither of these two variants form functional channels at the plasma membrane (Plummer et al., 1997). Instead, the Δ18 Na_**V**_1.6 channel isoform is expressed in vesicular intracellular compartments and crucially contributes in the control of podosome and invadopodia formation (Carrithers et al., 2009). In addition, *SCN5A* (Na_**V**_1.5) is expressed in the late endosome, rather than at the plasma membrane of the THP-1 cells. The intracellular Na_**V**_1.5 channel was shown to enhance endosomal acidification and phagocytosis (Carrithers et al., 2007), Ca^2+^ signaling, and phenotypic differentiation in human macrophages (Carrithers et al., 2011). The same group later demonstrated that *SCN5A* was expressed as a new splice variant lacking exon 25, resulting in a deletion of 18 amino acids in domain III (Rahgozar et al., 2013), generating non-selective outward currents and small inward currents in a heterologous expression system (Jones et al., 2014).

#### Na_V_α in Ewing sarcoma

The Ewing sarcoma (ES) is the second most common primary malignant bone tumor in children and adolescents, following osteosarcoma (Choi et al., 2014). RING1B, a member of the polycomb family of epigenetic regulators, is highly expressed in primary ES tumors. Depletion of RING1B with shRNA in ES cells enriched the expression of genes involved in hematological development, without affecting cellular differentiation (Hernandez-Munoz et al., 2016). Importantly, in ES cells, RING1B directly binds to the promoter of *SCN8A*, and its depletion results in enhanced Na_**V**_1.6 expression and function. In addition, the migratory speed of RING1B-depleted ES cells was attenuated, suggesting an inverse correlation between *SCN8A* expression and the migration capabilities of ES cells. Finally, reduced Na_**V**_1.6 function appeared to protect ES cells from apoptosis by a mechanism that maintains low NF-κB levels (Hernandez-Munoz et al., 2016). These findings revealed striking differences in the participation of *SCN8A* and its product, the Na_**V**_1.6 channel, in sarcomas compared with carcinomas and leukemia. Indeed, Na_V_1.6 appeared to have anti-cancer properties in ES, whereas it has pro-invasive functions in carcinomas and leukemia. Therefore, further studies are needed to fully understand the function of *SCN8A* across different types of cancer.

### Conclusions on the roles of Na_V_α and Na_V_β subunits in cancers

#### Pore-forming Na_V_α subunits

As previously indicated, the three main Na_**V**_α-encoding genes found to be upregulated in cancers are *SCN5A, SCN8A,* and *SCN9A,* which encode Na_**V**_1.5, Na_**V**_1.6, and Na_**V**_1.7, respectively. On the other hand, recent reports showed the downregulation of *SCN8A* and *SCN9A* genes in some cases. The molecular determinants explaining why these specific isoforms are overexpressed in cancers are not known and might be tissue specific. However, it is tempting to consider the deregulation in tumors of transcription factors that normally restrict the expression of a suite of genes associated with specific tissue functioning in adult tissues, such as the repressor element silencing transcription factor (REST) that restrict the expression of Na_V_α channels in excitable cells (Bruce et al., 2004; Chong et al., 1995) or other epigenetic regulations such as histone acetylation/deacetylation (performed by Histone Acetylases HAT and Histones Deacetylases HDAC, respectively), DNA, or histone methylation. Indeed, it was recently proposed that REST and HDAC2 play important role as epigenetic regulators and their inhibition in MCF-7 breast cancer cells enhanced the expression of Na_V_1.5 and promoted invasive capacities (Kamarulzaman et al., 2017). Nevertheless, it is interesting to notice that, when specifically studied in cancer cells, several neonatal splice variants of channels have been identified (Fraser et al., 2005; Lopez-Charcas et al., 2018; Baptista-Hon et al., 2014; Carrithers et al., 2009), thus supporting the common hypothesis of the re-expression of developmental genes in cancers.

These channels are present at the plasma membrane of cancer cells where sodium currents have been recorded. With the exception of Na_V_1.6 in Ewing sarcoma cells (Hernandez-Munoz et al., 2016), all Na_V_α isoforms have been shown to bear oncogenic properties, promoting cancer cell invasion *in vitro*, as well as other behaviors associated with metastasis, such as the acquisition of elongated and mesenchymal-like phenotypes, directed migration, proliferation, regulation of endocytosis, control of intracellular and perimembrane pH, and extracellular matrix degradation (Yang et al., 2012; Hernandez-Plata et al., 2012; Roger et al., 2003; Grimes et al., 1995; Fraser et al., 2005; Gillet et al., 2009; Mycielska et al., 2003; Djamgoz et al., 2001; Brisson et al., 2013). In addition, Na_V_α subunits promote tumor growth, invasion, and metastasis in *in vivo* rodent models (Nelson et al., 2015b; Driffort et al., 2014; Batcioglu et al., 2012; Yildirim et al., 2012). Comparative studies performed in different cancer types indicate the involvement of these Na_V_α isoforms in similar functional properties, arguing for isoform-independent signaling pathways. The activity of the channels at the plasma membrane appears to be critical. Indeed, Navα subunits are functionally active in cancer cell lines and primary tumor cells cultured *in vitro*, as well as in murine tumor xenograft tissue slices *in vivo* (Fraser et al., 2005; Roger et al., 2003; Hernandez-Plata et al., 2012; Nelson et al., 2015b), and their inhibition, using different drugs and small molecules such as TTX, ranolazine, phenytoin, Cn2 or PF-05089771, inhibits invasion (Nelson et al., 2015a, 2015b; Driffort et al., 2014; Batcioglu et al., 2012; Yildirim et al., 2012; Lopez-Charcas et al., 2018; Roger et al., 2003, 2007; Liu et al., 2019).

Importantly, the membrane potential (*V*_*m*_) of cancer cells is typically relatively depolarized compared with terminally differentiated non-cancer cells (Yang and Brackenbury, 2013). At this range of Vm, Na_V_α channels would be expected to be predominant in the inactivated state. However, in cancer cells the *V*_*m*_ is generally between −40 and −30 mV and is situated in a window of voltage that provides a small non-inactivating persistent Na^+^ current flowing into the cell, locally increasing intracellular Na^+^ concentration (Yang et al., 2012; Roger et al., 2003; Campbell et al., 2013). Recently a Na^+^-dependent intracellular signaling pathway, involving Salt-inducible kinase 1, has been proposed to account for pro-invasive effects of Na_V_α (Gradek et al., 2019).

The main role attributed to Na^+^ is to serve as a mere mediator of the membrane potential, in excitable as well as in non-excitable cells. It is also characterized to support ion (among which Na^+^/K^+^, Na^+^/Ca^2+^, Na^+^/K^+^/Cl^−^, Na^+^/HCO_3_^−^) exchanges and nutrient/metabolite transports across membranes (Na^+^/glucose for example). The role of second messenger is mostly attributed to the Ca^2+^ ion, for which a lot of specific probes and tools have been developed over the last 20 years. In contrast, no direct and specific biological sensors for Na^+^ have been identified, and tools to study Na^+^ evolution still lack sensitivity or dynamics. Yet, there is some evidence suggesting that Na^+^ could act as a second messenger *per se* and might regulate several important signaling pathways in normal cells. Indeed, recent data support a direct role of Na^+^ in controlling kinases activity (Jaitovich and Bertorello, 2010), membrane fluidity, and protein diffusion through an interaction with phospholipids (Hernansanz-Agustin et al., 2020) or to induce inflammatory stress (Amara et al., 2016). Therefore, this raises the possibility that Na^+^ could also serve as a second messenger in cancer cells to activate signaling pathways promoting aggressiveness. To further support this hypothesis, it is worth mentioning that early studies questioned the involvement of intracellular Na^+^ content and the consequences on malignant cell proliferation, invasive capacities, and the development of metastases (Cone, 1974). Indeed, much higher Na^+^ concentrations have been recorded in tumor cells, as compared with non-cancer cells by energy-dispersive X-ray microanalyses (Cameron et al., 1980) as well as by 23Na-magnetic resonance imaging (Ouwerkerk et al., 2007; Jacobs et al., 2004; Zaric et al., 2016) and was proposed to serve as an indicator of malignancy.

The inward Na^+^ current may also further depolarize the *V*_*m*_, which might also participate in promoting migration. In support of this hypothesis, it was demonstrated in breast cancer cells that Na_V_1.5 sustained *V*_*m*_ depolarization, which activated the RhoGTPase Rac1, subsequently inducing cytoskeletal reorganization and cellular migration (Yang et al., 2020) (Figure 2). Although there is clear evidence that Na_V_α channels expressed at the plasma membrane are critical in the acquisition of oncogenic properties, the discovery of splice variants with expression restricted to intracellular compartments, such as endosomes, phagosomes, or lysosomes (Lopez-Charcas et al., 2018; Carrithers et al., 2009), suggests a more complex role.

#### (More than) auxiliary Na_V_β subunits

The main Na_**V**_β-encoding gene found to be upregulated in cancers is *SCN1B,* encoding for the Na_**V**_β1 subunit (Diss et al., 2008; Nelson et al., 2014; Bon et al., 2016; Sanchez-Sandoval and Gomora, 2019). On the other hand, there are multiple reports showing the downregulation of *SCN4B* (Na_**V**_β4) (Hernandez-Plata et al., 2012; Bon et al., 2016; Diss et al., 2008; Gong et al., 2018; Sanchez-Sandoval and Gomora, 2019) and of *SCN3B* (Na_**V**_β3) in some instances (Bon et al., 2016; Adachi et al., 2004)*.*

The Na_**V**_β1 subunit has been shown to increase cancer proliferation, cell adhesion, increase neurite-like process outgrowth formation, and promote cancer cell invasion, while slowing migration *in vitro* (Nelson et al., 2014; Chioni et al., 2009; Bon et al., 2016; Sanchez-Sandoval and Gomora, 2019). *In vivo*, Na_**V**_β1 overexpression increases angiogenesis and reduces apoptosis, thus increasing tumor growth and metastasis (Nelson et al., 2014). Taken together, these results are in favor of a pro-cancerous role of Na_**V**_β1, and the effects appear to be dependent in part on the regulation of the Na_V_α pore-forming subunit as well as on the extracellular CAM motif (Nelson et al., 2014). Na_**V**_β2 expression in prostate cancer cells also increases process extension, adhesion, invasion, and migration *in vitro* but reduces tumor take *in vivo* (Jansson et al., 2012, 2014). Conversely, Na_**V**_β3 and Na_**V**_β4 may function as tumor suppressors. The *SCN3B* gene contains p53 response elements, and Na_**V**_β3 suppresses colony formation and promotes chemotherapy-induced apoptosis in a p53-dependent manner (Adachi et al., 2004). Na_**V**_ β4 expression is downregulated in breast, colorectal, lung, cervical, and prostate tumors and papillary thyroid cancer compared with normal tissue (Hernandez-Plata et al., 2012; Bon et al., 2016; Diss et al., 2008; Gong et al., 2018; Sanchez-Sandoval and Gomora, 2019). In addition, Na_V_β4 functions as a tumor and metastasis suppressor gene *in vivo* (Bon et al., 2016). This tumor-suppressing function occurs via β4-mediated control of RhoA GTPase activation (Bon et al., 2016) (Figure 2).

### Na_V_α as anticancer targets for repurposed drugs and new small inhibitory molecules

Na_V_α are attractive drug targets because of the broad therapeutic potential of their blockers. Considering the fact that Na_V_α are expressed in metastatic cells in various tumors, significant effort has been made to develop Na_V_α blockers as potential drugs for cancer treatment. This section of the review focuses on such efforts that took place in the past 10 years. These efforts for blocker development can broadly be classified into two sections: (1) repurposing drugs that are FDA approved for other clinical uses (local and general anesthetics, antiepileptic and anticonvulsant, antiarrhythmic drugs); (2) rational design and development of novel Na_V_α blockers for cancer treatment.

#### Repurposing of FDA-approved Na_V_α blockers

There are numerous existing Na_V_α inhibitors licensed for clinical use. In several cases Na_V_α inhibition is considered an off-target effect of these drugs. For example, tricyclic antidepressants, including amitriptyline, inhibit not only the serotonin transporter but also several neurotransmitter receptors and voltage-activated ion channels including Na_V_α. In other cases Na_V_α inhibition is considered the primary mechanism of the drug's intended therapeutic effect. This is true for several anti-seizure medications (e.g. phenytoin and carbamazepine) and all of the local anaesthetics, although these too have additional off-target actions. Regardless of whether it is a primary or secondary effect of a licensed medication, Na_V_α inhibition might be beneficial in patients with cancers associated with Na_V_α expression. This raises the intriguing possibility that approved Na_V_α-inhibiting drugs might be repurposed to treat cancer.

Benefits of repurposing approved medications include prior knowledge of their mechanisms of action and the availability of toxicology and safety data, thereby avoiding the need for drug discovery and early phase clinical trials. Drawbacks include limited potential for developing intellectual property, leading to a lack of both funding potential and industry involvement (Pushpakom et al., 2019). Nevertheless, despite the potential drawbacks, there are some notable successes, and a well-trodden pathway to repurposing is through the use of electronic health records to link prescribing data to potentially beneficial health outcomes. A good example of the impact of this type of retrospective clinical analysis is the identification of an association of aspirin use with reduced risk of colon cancer (Dube et al., 2007). Similar approaches are being used in studies exploring a possible relationship between Na_V_α inhibitors and outcomes in cancer patients.

#### Anti-seizure and class 1 antiarrhythmic Na_V_α inhibitors

A recent study, using retrospective clinical analysis to explore the possibility of a beneficial effect of Na_V_ inhibiting medications, examined several class 1 antiarrhythmic and antiseizure medications in patients with breast, bowel, or prostate cancer (Fairhurst et al., 2015). The combined analysis revealed that these medications (including class I antiarrhythmic drugs, lamotrigine, carbamazepine phenytoin, and valproate) were collectively associated with decreased median time to death compared with the control patient group, with significantly increased mortality in the drug group. This study clearly does not support the idea of repurposing antiseizure Na_V_α inhibitors in the treatment of breast, bowel, or prostate these cancers. However, as the authors pointed out, the causes of death were not available in the large primary care dataset, and co-morbidities were among the likely confounding factors. In many cases, patients treated with Na_V_α-inhibiting drugs will be suffering from life-threatening disorders such as epilepsy, and it is difficult to completely accommodate this confound in retrospective analyses.

#### Analgesic Na_V_α inhibitors

There has been considerable recent interest in the idea that anesthetics and analgesics used during surgical tumor excision might influence subsequent cancer recurrence. Surgery can cause the release of tumor cells into the circulation, and the number of postsurgical circulating cancer cells is known to be a negative prognostic indicator of disease-free survival (Yu et al., 2018). The perioperative period, i.e. immediately before, during, and after surgery, may therefore be an opportune time for interventions that inhibit the potential for metastatic invasion. A variety of drugs are typically administered during surgery including general anesthetics, analgesics, and anti-muscarinic and neuromuscular blockers. Some of these, such as inhalational general anesthetics, may have the potential to worsen outcomes by suppressing the immune response (Stollings et al., 2016). By contrast, local anesthetics may provide more favorable outcomes. Local anesthetics are often administered regionally to provide blockade of afferent nociceptive fibers entering the spinal cord. Several retrospective clinical studies suggest that regional analgesia during breast and prostate cancer surgery increases disease-free survival (Forget et al., 2019). The use of regional anesthesia diminishes or abolishes the need for general anesthetic during surgery. It was therefore initially hypothesized that the general anesthetic sparing effect of regional analgesia with local anesthetics accounts for the apparent beneficial effect (Sessler et al., 2008). However, a recent large prospective multicenter trial comparing outcomes after breast cancer surgery under inhalational anesthesia with or without paravertebral analgesia by ropivacaine or levobupivacaine revealed no difference in disease-free survival (Sessler et al., 2019).

Most of the ongoing clinical trials exploring the impact of anesthetic technique on cancer outcomes are predicated on the idea that the potential benefit of local anesthetics is conferred indirectly through their inhalational anesthetic sparing effect. However, it is possible that local anesthetics such as lidocaine, ropivacaine, and levobupivacaine provide a direct beneficial effect through Na_V_α inhibition (Baptista-Hon et al., 2014; Elajnaf et al., 2018). If this is the case, then a more direct approach for administering these drugs directly onto the tumor may prove to be beneficial. Lidocaine, in addition to being a local anesthetic, is also used intravenously as a class 1b antiarrhythmic agent and a circulating analgesic. Ongoing clinical trials will test whether lidocaine delivered directly onto breast tumors prior to excision or intravenously during the perioperative period for colon cancer surgery will prolong postoperative disease-free survival (NCT01916317, R.A Badwe, 2013; NCT02786329, D. Ionescu, 2016). We await the outcome of these trials with interest and note that there are several other approved Na_V_α-inhibiting drugs that should be examined in retrospective clinical studies for potential beneficial effects in cancer outcomes.

#### Rational design of small molecule Na_V_α blockers

Rational designing of Na_V_α inhibitors has been difficult because detailed structural information of drug-binding sites for this integral membrane protein were lacking until very recently. Therefore, early effort for the discovery of Na_V_α blockers mainly relied on strategies such as ligand-based drug design, natural-product-based drug design, *in silico* screening, and similarity searches. However, recent reports on the structures of human and bacterial Na_V_α and bound ligands shed light on their binding site (Cervenka et al., 2018; Nguyen et al., 2019; Pan et al., 2018; Payandeh et al., 2011; Shen et al., 2017, 2018, 2019). These reports will certainly aid in the structure-based design and discovery of Na_V_α blockers. A summary of the available reports on the identification and evaluation of Na_V_α blockers for potential use in cancer therapy follows.

**Figure 3:**
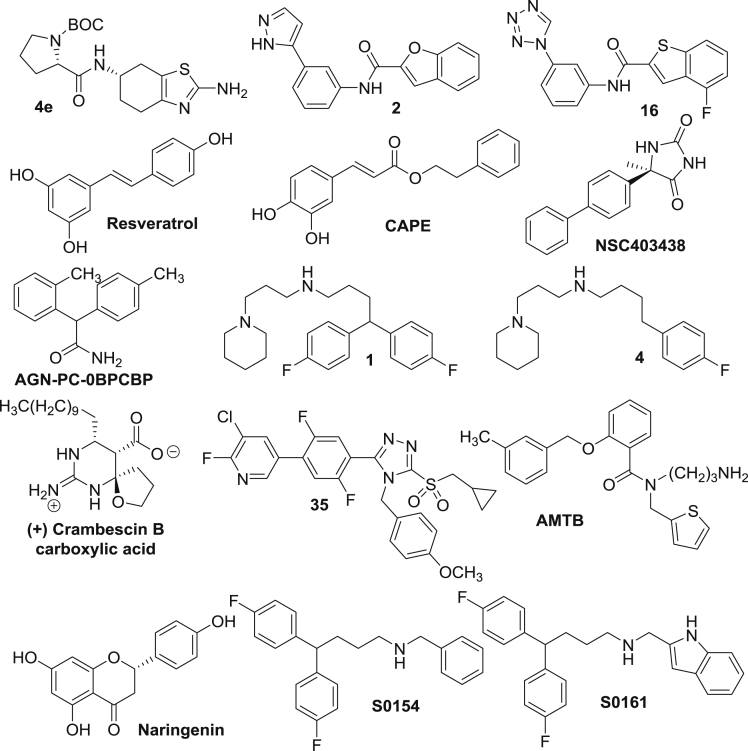
Chemical structures of known Na_V_α blockers with anticancer effects

One such effort to identify Na_V_α blockers used a pyrrole-imidazole marine alkaloid, clathrodin (Hodnik et al., 2013). Clathrodin was originally isolated from the *Agelas clathrodes* sponge. Several conformationally restricted analogues of clathrodin containing a 4,5,6,7-tetrahydrobenzo [d] thiazol-2-amine moiety are blockers of Na_V_1.3, Na_V_1.4, Na_V_1.5, and Na_V_1.7 channels. These compounds display state-dependent inhibitory activity of these channels at low micromolar concentrations. The most active compound (4e, Figure 3) identified from this study represents a novel selective blocker of Na_V_1.4 channel with an IC_50_ value of 8 μM. The use of clathrodin analogues as a template for ligand-based virtual screening of commercially available ZINC library of compounds using ROCS software identified two potent lead compounds 2 and 16 (Tomasic et al., 2013) (Figure 3). These blocked I_Na_ produced by Na_V_1.7 with IC_50_ values of 7 μM and 9 μM, respectively.

Plant-derived polyphenolic natural products have also been reported as Na_V_α inhibitors. For example, the plant phenolic, resveratrol (Figure 3), found at high concentrations in red grapes inhibits Na_V_α with an IC_50_ value of 50 μM (Fraser et al., 2014). Resveratrol also suppresses lateral cell motility by up to 25%, transverse cell motility by 31%, and cell invasion by 37%, without affecting cellular proliferation or cell viability of MAT-LyLu cells. Another similar phenolic is caffeic acid phenethyl ester (CAPE, Figure 3) isolated from honeybee propolis. CAPE blocks Na_V_ activity in several invasive cancer cell lines, including breast (MDA-MB-231 and MDA-MB-468), colon (SW620), and non-small cell lung cancer (H460). Motility and invasion of MDA-MB-231 cells were reduced by up to 14% and 51%, respectively by CAPE at 1 μM without affecting cell proliferative activity (Fraser et al., 2016).

Shaheen et al. used an *in silico* approach to identify Na_V_α blockers with superior pharmacological profile compared with phenytoin (PHT) and carbamazepine (CBZ) in 2015 (Shaheen et al., 2015). They conducted a similarity search in the PubChem database with PHT and CBZ as query molecules using the Tanimoto-based similarity search. The search was further refined by docking of these molecules into the binding site of the homology model of *SCN1A* using MolDock program. This study identified high-affinity compounds similar to PHT and CBZ. The lead compounds were further evaluated for toxicity profiles and biological activity. Two of the best compounds identified by this study, NSC403438 and AGN-PC-0BPCBP (Figure 3), demonstrated better binding affinity to Na_V_α compared with PHT and CBZ, with NSC403438 being a superior inhibitor of I_Na_ with lower toxicity, better IC_50_ value, and optimal bioactivity.

Na_V_α inhibitors were also derived from the natural product, crambescin (Nakazaki et al., 2016). Enantiomerically pure crambescin A, B, and C carboxylic acid derivatives were synthesized and evaluated for their ability to block Na_V_α. Structure activity relationship studies revealed that the natural enantiomer of crambescin B, carboxylic acid (Figure 3), is the most active compound with activity comparable to TTX. The cyclic guanidinium moiety present in this molecule is indispensable for its activity.

In 2018, Dutta et al. utilized a highly predictive, comprehensive 3D-QSAR model for the design of Na_V_α blockers (Dutta et al., 2018). The Na_V_α-binding data (IC_50_) for 67 compounds were used to train a comprehensive CoMFA model, which effectively covered 3D space and spanned over 4 orders of magnitude in biological activity. Potency predictions by this model have been highly accurate for more than 30 compounds that were synthesized and evaluated. Five compounds shown or predicted to have low nanomolar Na_V_α binding were further evaluated for the inhibition of hNa_V_1.5e currents in individual breast cancer MDA-MB-231 cells. Of these, two lead compounds, 1 and 4 (Figure 3), were found to be most effective in whole-cell patch-clamp studies and showed significant invasion inhibitory activities at concentrations as low as 1 μM without affecting cell viability.

Boezio et al. reported several sulfonamides with highly selective Na_V_1.7 blockade activity (Boezio et al., 2018). This novel series of blockers contained a triazole sulfone, which served as a bioisostere for the acyl sulfonamide group. This work resulted in the discovery of a series of potent Na_V_1.7 blockers with selectivity over Na_V_1.5 and favorable pharmacokinetic properties in rodents. An example of such a blocker is compound 35 (Figure 3). In the same year, Yapa et al. reported a known inhibitor of TRPM8, N-(3-aminopropyl)-2-{[(3-methylphenyl) methyl]oxy}-N-(2-thienyl methyl)benzamide (AMTB, Figure 3), as a Na_V_α blocker in breast cancer MDA-MB-231 cells (Yapa et al., 2018). AMTB decreased viable cell number in MDA-MB-231 and SK-BR-3 breast cancer cell lines and also reduced the migration of MDA-MB-231 cells. These studies provided the evidence that these effects are not related to TRPM8 inhibition but rather caused by the Na_V_α blockade caused by AMTB. Gumushan Aktas et al. investigated the potential effects of a natural flavanone, naringenin (Figure 3), on the motility of MAT-LyLu cells in 2018 (Gumushan Aktas and Akgun, 2018). This study revealed that naringenin inhibited cell proliferation at higher concentrations (75 μM), whereas it decreased the movement of MAT-LyLu cells at low concentrations (5 μM and 10 μM). Moreover, naringenin inhibited cell motility by reducing the expression of the *SCN9A* gene at the mRNA level. In conclusion, naringenin was found to have direct or indirect blocking activity on the *SCN9A*-encoded channel. Most recently, in 2019, Wang et al. has reported Na_V_1.7 channel blockers using a comparative molecular field analysis (CoMFA) model for the binding of ligand to Na_V_α, generated based on diverse set of compounds. No channel current blockade data were presented in the paper. However, there was an extensive anticancer evaluation of the identified lead compounds S0154 and S0161 (Wang et al., 2019). Both showed anticancer and anti-metastatic effects against PC3 prostate cancer cells and significantly inhibited cell viability, with IC_50_ values in the range of 5–26 μM. Both these compounds inhibited the expression of Na_V_α, increased the intracellular level of Na^+^, and caused cell-cycle arrest in G2/M phase. The compounds also inhibited the invasion of PC3 cells. Furthermore, S0161 inhibited the PC3 tumor growth by about 51% in an *in vivo* xenograft model (Wang et al., 2019).

In conclusion, there have been major advances in Na_V_α-targeted drug discovery over the last decade. Specific Na_V_α isoforms have been implicated in the metastasis development of a variety of tumors raising the possibility of developing tumor selective drugs. Recent advances in the discovery of high-resolution crystal and cryoEM structures of Na_V_α should further advance the field with structure-based drug design efforts.

### Na_V_α as targets for nutritional management of cancers

Cancer is a metabolic disease that depends on bioenergetic parameters (Penkert et al., 2016). Cancer progression is generally associated with the survival of cancer cells under conditions of low oxygen levels and nutrient deprivation and relies on metabolic adaptations (Dumas et al., 2017; Hanahan and Weinberg, 2011). These metabolic adaptations allow cancer cells to survive the pressure of environmental conditions and to fulfill the high energy demands associated with their high anabolic activity (Payen et al., 2016; Porporato et al., 2016). Also, this metabolic switch toward an aerobic glycolysis brings selective advantages by promoting invasive activities and metastatic properties (Brisson et al., 2012; Webb et al., 2011). Furthermore, the metabolic reprogramming concerns not only tumor cells but also multiple cell types and organs of the host, thus leading to an overall deregulation of the energetic balance in patients, called tumor cachexia (Fearon et al., 2012). This devastating syndrome, initially triggered by the release of soluble tumor factors and the participation of a systemic inflammation, is characterized by anorexia, the loss of skeletal muscle mass, in some cases with the loss of adipose tissue mass, and a general weakening of patients, impeding their quality of life and decreasing the tolerance to antineoplastic therapies (Fonseca et al., 2020; Biswas and Acharyya, 2020). In this context, bringing a nutritional support to patients is required to allow holding the most efficient possible treatment. Nutritional interventions are mostly aimed at preventing the wasting of body compartments in patients but could also be a source of active anti-cancer molecules. Furthermore, diet represents a controllable component of the environment and brings promising strategies to increase treatment efficacy in combination with conventional chemotherapeutics. Some dietary compounds have also been shown to decrease the risk of carcinoma development and may prolong the survival of patients (Dumas et al., 2017).

As pointed out in the previous section, several natural compounds in the diet, such as resveratrol and caffeic acid phenethyl esters, are effective at inhibiting Na_V_α subunits (Fraser et al., 2014, 2016). Dietary lipids have also been proposed to inhibit Na_V_α and modulate cancer progression. Indeed, dietary lipids incorporate into cellular membrane and alter Na_V_α or their pharmacology (Agwa et al., 2018; D'avanzo, 2016). Among dietary lipids, long-chain n-3 polyunsaturated fatty acids (n-3 PUFA) have been described in epidemiological studies to delay or prevent the appearance of breast cancer (Rose et al., 1996; Bougnoux et al., 2010). From both *in vivo* and *in vitro* studies, n-3 PUFA have been reported to induce multiple anti-tumour effects, and their dietary consumption was associated with a lower risk of cancers, such as breast or colorectal cancers (Bougnoux, 1999; Bougnoux et al., 2010; Eltweri et al., 2016). Even though n-3 PUFA were suggested to prevent prostate cancer (Moreel et al., 2014; Li et al., 2017), their beneficial effect remains to be demonstrated through more intervention trials or observational studies (Aucoin et al., 2016). A pilot study showed that fatty acid composition of breast adipose tissue differed according to breast cancer focality: low levels of the two long-chain n-3 PUFA docosahexaenoic acid (DHA, 22:6n-3) and eicosapentaenoic acid (EPA, 20:5n-3) were associated with tumor multifocality, which is considered a marker of cancer aggressiveness (Ouldamer et al., 2016a). These results could indicate that differences in adipose tissue concentration, a surrogate of past dietary uptake, may contribute to mechanisms influencing cancer progression.

Long-chain n-3 PUFA have been proposed to increase tumor sensitivity to chemotherapeutic agents with no sensitization of normal tissues and no additional side effect (Bougnoux et al., 2010). As such, DHA and EPA have generated intense interest due to their ability to reduce resistance to anthracyclines, taxanes, or radiotherapy in mammary tumor models (Bougnoux et al., 2010; Hajjaji et al., 2011, 2012; Ouldamer et al., 2016b).

N-3 PUFA have also been shown to have anti-invasive and anti-metastatic properties (Blanckaert et al., 2010; Gillet et al., 2011; Rose et al., 1994, 1997; Bougnoux et al., 2010). On the other hand, they are capable of modulating the activity of NHE exchangers (Besson et al., 1996; Lacroix et al., 2008) and ion channels (Tillman and Cascio, 2003; Jude et al., 2003). Interestingly, in expression systems and in native rat cardiomyocytes, the activity of Na_V_1.5 was initially found to be inhibited by n-3 PUFA (Kang and Leaf, 1996; Kang et al., 1997) and in initial studies have proposed that these effects could be mediated by a direct binding of n-3 PUFA to specific residues of the channel (Xiao et al., 1998, 2001). Therefore, n-3 PUFA could also exert their beneficial effects on cancers through a reduction of Na_V_1.5 (Pignier et al., 2007; Gillet et al., 2011). However, contrasting results were obtained in human breast cancer cells in which I_Na_ was not inhibited by acute applications of n-3 PUFA, even at high concentrations (30–50 μM) (Wannous et al., 2015) at which they also have anti-proliferative effects (Barascu et al., 2005). This discrepancy might be due to the fact that cancer cells mostly express the hNa_V_1.5e neonatal splice variant (Fraser et al., 2005). However, growing breast cancer cells in the presence of low doses of DHA (0.5–10 μM) reduced *SCN5A* gene expression and levels of Na_V_1.5 proteins and I_Na_ (Wannous et al., 2015; Isbilen et al., 2006). This inhibition of *SCN5A* expression was mediated by the lipid-sensitive nuclear receptor peroxisome proliferator-activated receptor β (PPAR-β). Correlatively, the inhibition of Na_V_1.5 activity was also responsible for a reduced activity of the downstream protagonist NHE-1, thus decreasing H^+^ efflux, preventing extracellular matrix degradation proteolytic activity, and inhibiting breast cancer cell invasiveness (Wannous et al., 2015). A recent report also demonstrated the efficacy of EPA to reduce migration and proliferation of ovarian cancer cells by inhibiting Na_V_1.5 (Liu et al., 2018).

Such regulations concerning other Na_V_α involved in cancer properties, i.e. Na_V_1.6 and Na_V_1.7, should be investigated. It is also of interest to note that n-3 PUFA, through the activation of PPAR-γ, have been shown to downregulate the expression of NHE-1 and reduce cancer colony growth (Kumar et al., 2009). Furthermore, incorporation of n-3 PUFA into phospholipids induces changes in the physico-chemical properties of cell membranes (Yaqoob and Shaikh, 2010), which in turn affects NHE-1 activity (Dendele et al., 2014).

Further to these effects on Na_V_ channels and downstream signaling pathways, it should be mentioned that n-3 PUFA might exert a multiplicity of actions by interfering with several signaling pathways, some of them being beneficial to the prevention or treatment of cancer. As such, a lack of specificity is not obligatorily detrimental, and pleiotropic effects might increase the efficacy of the anticancer treatment.

N-3 PUFA supplementations were proposed to have beneficial effects in reducing mammary tumor growth, by slowing down cancer cell proliferation (Barascu et al., 2005). N-3 PUFA treatment was demonstrated to inhibit cyclin B1 and the expression of the cell division cycle 25C phosphatase, which dephosphorylates cyclin-dependent kinase 1 (Barascu et al., 2005). In addition, the nuclear receptor PPARβ appeared to regulate the DHA-related inhibition of MDA-MB-231 and MCF-7 cells proliferation. This allowed identifying PPARβ as an important protagonist in the inhibition of breast cancer cell proliferation and mammary tumor growth under DHA-enriched diet (Wannous et al., 2013). N-3 PUFA have been proposed to regulate autophagy in cancer cells and as such could be involved in both survival and apoptosis, depending on the carcinogenetic phase and the treatment context (Ferro et al., 2020). DHA was found to induce apoptosis in cancer cells (Jing et al., 2011). DHA-induced autophagy was associated with p53 loss, with the activation of AMPK and the decrease in the activity of mTOR. Autophagy inhibition suppressed apoptosis, and autophagy induction further enhanced apoptosis in response to DHA treatment (Jing et al., 2011). There is evidence that n-3 PUFA may inhibit the expression of EMT markers and reduce associated invasive properties in cancer cells (D'eliseo et al., 2016; Yin et al., 2016). Altogether, these effects could bring beneficial values to delay the appearance/diagnosis of a primary tumor and as such be of interest in primary prevention.

N-3 PUFA, which are highly peroxidizable, were also proposed to improve the efficacy of anticancer treatments by amplifying oxidative stress generated by anthracyclines or radiotherapy (Bougnoux et al., 2009). The acquisition of resistance to chemotherapeutic agents represents an important limitation in cancer. Resistance to taxanes has been proposed to depend on the induction of signaling pathways such as PI3K/Akt and ERK1/2, which promote survival and cell growth in human cancer cells. In docetaxel-treated MDA-MB-231 cells, phosphorylated-ERK1/2 levels were increased by 60% in both membrane and nuclear compartments, compared with untreated cells, and ERK1/2 activation depended on PKCε and PKCδ activation. In comparison, in cells treated with DHA, docetaxel was unable to increase PKCε and PKCδ levels, thus resulting in the reduction of ERK1/2 phosphorylation and the increase in docetaxel efficacy (Chauvin et al., 2016). In addition to these effects, n-3 PUFA were proposed to increase the efficacy of the chemotherapeutic treatment by remodeling the tumor vascular network, thereby improving the delivery of anticancer drugs within the tumor (Kornfeld et al., 2012). These results support the hypothesis that n-3 PUFA, which do not induce any toxic effect, could be used as an adjuvant to cancer therapy.

### Conclusions and perspective for the treatment of cancers

There is now clear evidence that the abnormal expression of Na_V_ subunits occurs during carcinogenesis and is associated with cancer progression toward metastatic states. Several different Na_V_α play a role, including Na_V_1.5, Na_V_1.6, and Na_V_1.7, but the intracellular signaling pathways they regulate appear to be the same or very similar in all studied cancer types, leading to the induction of invasive properties. The activity of such channels at the plasma membrane, and consequent I_Na_, appear critical. Therefore, the inhibition of Na_V_α in cancer cells represents a new anti-cancer strategy that could be achieved in several ways alone or in combination: repurposing existing Na_V_α-inhibitory drugs, developing new small inhibitory molecules, and through dietary interventions. As shown above, Na_V_β subunits also have important roles in cancer cell biology and in cancer progression, acting both as auxiliary subunits of Na_V_α and as CAMs. However, they do not exert a recordable activity *per se*, and their direct “inhibition” or “activation” would be challenging. Therefore, dietary strategies aiming at controlling their expression, i.e. reducing the expression of *SCN1B* and *SCN2B*, while maintaining the expression of *SCN3B* and *SCN4B*, might represent powerful strategies. For this purpose, future studies aiming at unraveling transcriptional and epigenetic regulators will be of high interest.
